# Cytomegalovirus, Epstein‐Barr virus, and human herpesvirus‐6 infections in patients with myalgic еncephalomyelitis/chronic fatigue syndrome

**DOI:** 10.1002/jmv.25744

**Published:** 2020-03-11

**Authors:** Evelina Shikova, Valentina Reshkova, Аntoniya Kumanova, Sevdalina Raleva, Dora Alexandrova, Natasa Capo, Modra Murovska, on behalf of the European Network on ME/CFS (EUROMENE)

**Affiliations:** ^1^ Department of Virology National Center of Infectious and Parasitic Diseases Sofia Bulgaria; ^2^ The National Specialized Hospital for Active Treatment in Haematological Diseases Sofia Bulgaria; ^3^ University Hospital “St Ivan Rilski,” Sofia Bulgaria; ^4^ Oncology Institute of Vojvodina Sremska Kamenica Serbia; ^5^ Faculty of Medicine University of Novi Sad Novi Sad Serbia; ^6^ Institute of Microbiology and Virology Riga Stradins University Riga Latvia

**Keywords:** active infection, CMV, EBV, HHV‐6 infection, ELISA, myalgic encephalomyelitis/chronic fatigue syndrome (ME/CFS), PCR

## Abstract

Myalgic encephalomyelitis/chronic fatigue syndrome (ME/CFS) is a disabling multisystem chronic disease. The etiology and pathogenesis of ME/CFS are unknown. Infections of cytomegalovirus (CMV), Epstein‐Barr virus (EBV), and human herpesvirus‐6 (HHV‐6) are suspected as etiological agents for ME/CFS. This study aims to estimate prevalence and type (active/latent) of EBV, CMV, and HHV‐6 infections in Bulgarian ME/CFS patients. In the study were included 58 patients with ME/CFS and 50 healthy controls. Virus‐specific antibodies were detected by enzyme‐linked immunosorbent assay and viral genomic sequences in peripheral blood mononuclear cell (PBMCs) and plasma samples by nested polymerase chain reaction (PCR). We did not observe any significant differences in virus‐specific immunoglobulin G and immunoglobulin M positivity rates between patients with ME/CFS and control group. In ME/CFS plasma samples, EBV DNA was found in 24.1%, CMV DNA in 3.4%, and HHV‐6 DNA in 1.7% of samples. EBV DNA was detected in 4%, and CMV and HHV‐6 DNA were not found in plasma samples of controls. The frequency of viral genome detection in PBMCs of patients and controls was 74% vs 78% for CMV, 81% vs 84% for EBV, and 82.8% vs 82% for HHV‐6. The difference in frequency of EBV active infection in ME/CFS and control group was statistically significant (*P* = .0027). No ME/CFS and control individuals with active CMV and HHV‐6 infection were observed. In conclusion, this study using both serological and PCR‐based techniques for distinguishing between active and latent infection showed high rate of active EBV infection among patients with ME/CFS indicating that at least in a subset of cases, EBV is important factor for the development of disease.

## 1 INTRODUCTION

Myalgic encephalomyelitis/chronic fatigue syndrome (ME/CFS) is a disabling multisystem chronic disease. The main clinical sign is debilitating persisting chronic fatigue, not relieved by rest. In addition to fatigue, patients with ME/CFS also suffer from a variety of other symptoms including postexertional malaise, cognitive impairment, musculoskeletal pain, sleep dysfunction, sore throats, lymphadenopathy, orthostatic intolerance, and gastrointestinal symptoms. The disease is poorly understood and no diagnostic biomarkers are currently available. Therefore, the diagnosis of ME/CFS is difficult and requires exclusion of other medical conditions. It is based on several different sets of diagnostic criteria/case definitions, of which the most widely used are Fukuda case definition, Canadian consensus criteria, and International Consensus Criteria.^1^, ^2^, ^3^ The etiology and pathogenesis of ME/CFS are still unknown. Dysregulation of immune system, autonomic nervous system, and metabolic disturbances are the most popular explanatory models for ME/CFS.^4^, ^5^, ^6^, ^7^, ^8^ The hypotheses for etiology include genetic predisposition, immune dysfunction, infectious agents, metabolic disturbances, brain dysfunction, toxins, stress, trauma, circulatory abnormalities, or a combination of any of these factors. As in many patients with ME/CFS, the disease starts suddenly with a “flu‐like’’ illness, it was suggested that an infectious agent can trigger the syndrome. Numerous viruses have been associated with the development of ME/CFS including enteroviruses, herpesviruses, retroviruses, parvovirus B19, hepatitis C virus, Ross River virus (RRV).^8^, ^9^, ^10^, ^11^, ^12^, ^13^, ^14^, ^15^ It was shown that the severity of acute Epstein‐Barr virus (EBV) and RRV infection and the host response may determine the course of postinfectious fatigue and ME/CFS and was suggested that inflammatory cytokines influence the CNS, resulting in neurocognitive disturbances.^16^, ^17^ In addition, according to Duvignaud et al,^18^ CFS‐like illness may develop in 26% of patients with chronic fatigue as a result of postchikungunya chronic disease, induced by chikungunya virus. However, although the correlation between viral infections and ME/CFS has been studied for a long time, the role of viruses in the etiology of ME/CFS is still uncertain.

Herpesviruses have frequently been associated with ME/CFS. Infections with EBV, human herpesvirus‐6 (HHV‐6), and cytomegalovirus (CMV) are considered as triggering factors for ME/CFS.^9^ After an acute infection, these viruses persist life‐long in various cells of the body and may reactivate. There are hypotheses that the reactivation of a latent virus could damage the immune system and contribute to the morbidity of ME/CFS. Another possibility is that patients with ME/CFS are susceptible to acute viral infections as a consequence of immune dysfunction. At the same time, these viruses are ubiquitous in the general population and, therefore, it is difficult to prove their causative roles.

Despite multiple studies on the association of EBV, CMV, and HHV‐6 with ME/CFS, the data are not consistent. This study aims to estimate the prevalence and type of EBV, CMV, and HHV‐6 infections in Bulgarian patients with ME/CFS using both serological and polymerase chain reaction (PCR)‐based techniques.

## 2 MATERIALS AND METHODS

### 2.1 Study participants

A total of 108 subjects were recruited for this study—58 patients with ME/CFS and 50 healthy persons as a control group. The patients were diagnosed with ME/CFS according to Fukuda criteria.^1^ They were aged between 19 and 60 years (average 39 years) and women were more prevalent (72%) than men (28%). The control group included 34 females and 16 males with average age of 42 years.

### 2.2 Ethical issues

This study has been approved by the Ethical Committee of the National Center of Infectious and Parasitic Diseases, Sofia, Bulgaria. All participants provided informed written consent before their enrollment.

### 2.3 Sample collection and processing

Blood samples were collected in ethylenediaminetetraacetic acid (for peripheral blood mononuclear cells [PBMCs] and plasma) and Gel/Clot Activator (for serum) vacutainers. PBMCs were prepared from whole blood by Histopaque‐1077 (Sigma‐Aldrich) density gradient separation. Serum samples were used for enzyme‐linked immunosorbent assay (ELISA) testing. DNA was isolated from PBMCs and blood plasma samples.

### 2.4 Enzyme‐linked immunosorbent assay

ELISA testing was used to detect immunoglobulin G (IgG) and immunoglobulin M (IgM) class antibodies specific to CMV, EBV, and HHV‐6. All serum samples were tested by using commercial ELISA kits for the following virus‐specific antibodies: CMV IgM and IgG, EBV capsid antigen (CA) IgM and IgG, (EUROIMMUN, Medizinische Labordiagnostika AG, Lubeck, Germany); HHV‐6 IgG and IgM (VIDIA, Vestec, Czech Republic). The EBV‐CA positive samples were further tested for the presence of EBV nuclear antigen 1 (EBNA‐1) IgG (EUROIMMUN, Medizinische Labordiagnostika AG). Performance and interpretation of results of all tests were done according to the manufacturer's instructions. Each ELISA was run with negative and positive controls, and calibrator (cutoff). For each measurement of an antibody concentration, a ratio (R) (EUROIMMUN) and index value (Iv) (VIDIA) were calculated as follows: sample optical density (OD) value was divided by cutoff OD value. Serum samples with R ≥ 1.1 (EUROIMMUN) and Iv > 1 (VIDIA) were regarded as positive. Test results with R/Iv > 5.0 were for the purpose of this study considered as highly positive. Samples with equivocal results were retested.

### 2.5 Nested polymerase chain reaction

DNA was isolated from PBMCs using PureLink Genomic DNA Mini Kit (Invitrogen) and from cell‐free blood plasma by PureLink Viral RNA/DNA Mini Kit (Invitrogen) according to the manufacturer's instructions. The quality of the PBMCs DNA and the absence of contamination of plasma DNA by cellular DNA were evaluated by β‐globin gene amplification as previously described.^19^ Nested polymerase chain reaction (nPCR) was used to amplify specific CMV, EBV, and HHV‐6 DNA sequences in PBMCs and cell‐free blood plasma from patients with ME/CFS and healthy controls. Detection of CMV, EBV, and HHV‐6 DNA by nPCR was mainly based on previous studies of Allen et al,^20^ Cinque et al,^21^ and Secchiero et al,^22^ respectively. The reaction volume of the first and second PCR was 50 μL and included AmpliTaq Gold 360 Master Mix (Applied Biosystems), 10 pmol of each primer, nuclease‐free water, and 10 μL of template. The primers used for amplification of CMV, EBV, and HHV‐6 DNA sequences are described in Table 1. They targeted conserved regions of virus genome: glycoprotein H gene of CMV, main capsid protein gene of HHV‐6, and EBNA‐1 of EBV. Positive and negative controls were included in each experiment. DNA positive controls for CMV and EBV were purchased from Genekam Biotechnology AG (Duisburg, Germany) and for HHV‐6 was a gift from Prof M. Murovska (Institute of Microbiology and Virology, Riga Stradiņš University, Latvia). As negative controls DNA from CMV, EBV, and HHV‐6 negative individuals, as well as water controls, were used. The cycling conditions were as follows: for both cycles of CMV—initial denaturation at 94°C for 3 minutes, followed by 40 cycles (94°C/30 seconds, 56°C/30 seconds, and 72°C/45 seconds) and final extension for 5 minutes at 72°C; for both cycles of HHV‐6—initial denaturation at 94°C for 3 minutes, followed by 30 cycles (94°C/1 minute, 57°C/1 minute, and 72°C/1 minute) and terminal extension for 5 minutes at 72°C; first cycle of EBV—initial denaturation for 3 minutes at 94°C, 40 cycles (94°C/30 seconds, 55°C/30 seconds, and 72°C/45 seconds), terminal extension for 5 minutes at 72°C; during the second cycle of EBV DNA amplification, the annealing temperature was 60°C.

**Table 1:** Primers used in nPCR assays

Primers	Primer sequences (5′‐3′)	Amplicon, bp	Target region
CMV			Glycoprotein H gene
External primers	TGGACCTGGCCAAACGAGCCC	205	
	TGGACGAGGCTGCCCATGAGG		
Internal primers	TCACCGACATCACCAGCCTCG	159	
	CTTGGCGCGCGAAGGCTGAAAG		
EBV			EBNA‐1 gene
External primers	AAGGAGGGTGGTTTGGAAAG	297	
	AGACAATGGACTCCCTTAGC		
Internal primers	ATCGTGGTCAAGGAGGTTCC	209	
	ACTCAATGGTGTAAGACGAC		
HHV‐6			Main capsid protein gene
External primers	GCGTTTTCAGTGTGTAGTTCGGCAG	520	
	TGGCCGCATTCGTACAGATACGGAGG		
Internal primers	GCTAGAACGTATTTGCTGCAGAACG	258	
	ATCCGAAACAACTGTCTGACTGGCA		

Abbreviations: CMV, cytomegalovirus; EBNA‐1, EBV nuclear antigen 1; EBV, Epstein‐Barr virus; HHV‐6, herpesvirus‐6; nPCR, nested polymerase chain reaction.

PCR products were analyzed by electrophoresis in 2% agarose gels, stained with ethidium bromide and observed under UV light. A result was considered positive when both the sample and positive control in the second round of PCR presented a band corresponding to 159‐bp (CMV), 209‐bp (EBV), and 258‐bp (HHV‐6) DNA fragment, while there was no band in the negative control. All virus‐positive plasma samples were retested.

### 2.6 Quantitative real‐time PCR

The viral load of EBV in plasma samples from ME/CFS patients with active viral infection was determined using Sacace EBV Real‐TM Quant Kit, based on EBV LMP‐gene DNA amplification (Sacace Biotechnologies Srl, Como, Italy) according to the manufacturer's instructions. The amplifications were carried out in Exicycler 96 thermocycler from Bioneer (Bioneer Corp, South Korea).

### 2.7 Statistical analysis

Analysis of data was performed with SPSS for Windows v.10.0. Fisher's exact test was used to test for a statistically significant difference in the frequency of positivity of virus‐specific markers, as well of active and latent CMV, EBV, and HHV‐6 infection between ME/CFS patient group and control group. *P < *.05 were considered statistically significant.

## 3 RESULTS

### 3.1 CMV, EBV, and HHV‐6 serology

All serum specimens were tested for the presence of serum antibodies (both IgG and IgM) against CMV, EBV‐CA, and HHV‐6 by ELISA. The data are presented in Table 2. Specific anti‐CMV IgG antibodies were detected in 86.2% (50/58) and anti‐CMV IgM in 5.2% (3/58) of ME/CFS serum samples and in 88% (44/50) and 2% (1/50) of control samples, respectively. Anti‐EBV‐CA IgG antibodies were found in 96.6% (56/58), anti‐EBV‐CA IgM in 8.6% (5/58) of serum samples from ME/CFS cases. In the control group, we found 98% (49/50) EBV‐CA IgG positivity, none of the serum samples was positive for EBV‐CA IgM. In the ME/CFS group, 96.6% (56/58) and 8.6% (5/58) of serum samples were positive for HHV‐6 IgG and IgM, respectively. In the control group, 98% (49/50) of samples were IgG positive and 6% (3/50) were IgM positive. All CMV, EBV, and HHV‐6 IgM positive samples were also IgG positive. All EBV‐CA positive cases were also positive for EBNA‐1 IgG antibodies.

**Table 2:** CMV, EBV, and HHV‐6 serology determined by ELISA

	CMV		EBV‐CA		HHV‐6	
	IgG positive	IgM positive	IgG positive	IgM positive	IgG positive	IgM positive
	n (%)	n (%)	n (%)	n (%)	n (%)	n (%)
Patients with ME/CFS n = 58	50 (86.2%)	3 (5.2%)	56 (96.6%)	5 (8.6%)	56 (96.6%)	5 (8.6%)
Controls n = 50	44 (88%)	1 (2%)	49 (98%)	0	49 (98%)	3 (6%)
*P*	1.0	.6222	1	.0601	1.0	.7224

Abbreviations: CMV, cytomegalovirus; EBV‐CA, Epstein‐Barr virus capsid antigen; ELISA, enzyme‐linked immunosorbent assay; HHV‐6, herpesvirus‐6; IgG, immunoglobulin G; IgM, immunoglobulin M; ME/CFS, myalgic encephalomyelitis/chronic fatigue syndrome.

For all three viruses (CMV, EBV, and HHV‐6), we did not observe any statistically significant differences in IgG and IgM positivity rates between ME/CFS patients and control group (*P* = .0601‐1, for tested serological markers).

### 3.2 Detection of viral DNA by nPCR

#### 3.2.1 CMV, EBV, and HHV‐6 genomic sequences in plasma samples

Of all 58 tested plasma samples from patients with ME/CFS, CMV DNA was found in 3.4% (2/58), EBV DNA in 24.1% (14/58), and HHV‐6 DNA in 1.7% (1/58) of the samples (Table 3). EBV DNA was detected in 4% (2/50) of control plasma samples. CMV and HHV‐6 DNAs were not found in healthy individuals. The difference in EBV DNA detection in plasma samples of ME/CFS and control groups was statistically significant (*P* = .0052). There was no statistically significant difference for CMV (*P* = .4981) and HHV‐6 (*P* = 1.00).

**Table 3:** EBV, CMV, and HHV‐6 DNA in plasma and PBMCs samples

	CMV DNA positive		EBV DNA positive		HHV‐6 DNA positive	
	Plasma	PBMCs	Plasma	PBMCs	Plasma	PBMCs
	n (%)	n (%)	n (%)	n (%)	n (%)	n (%)
Patients with ME/CFS n = 58	2 (3.4%)	43 (74.1%)	14 (24.1%)	47 (81.0%)	1 (1.7%)	48 (82.8%)
Controls n = 50	0 (0%)	39 (78%)	2 (4%)	42 (84%)	0 (0%)	41 (82%)
*P*	.4981	.6597	.0052	.8018	1	1

Abbreviations: CMV, cytomegalovirus; EBV, Epstein‐Barr virus; ELISA, enzyme‐linked immunosorbent assay; HHV‐6, herpesvirus‐6; ME/CFS, myalgic encephalomyelitis/chronic fatigue syndrome; PBMC, peripheral blood mononuclear cell.

#### 3.2.2 EBV, CMV, and HHV‐6 DNA in PBMCs

CMV DNA was found in 74.1% (43/58), EBV DNA in 81% (47/58), and HHV‐6 DNA in 82.8% (48/58) of the PBMCs samples from patients with ME/CFS. In PBMC DNA samples of the control group, CMV, EBV, and HHV‐6 DNA were detected in 78% (39/50), 84% (42/50), and 82% (41/50), respectively (Table 3). There was no statistically significant difference between ME/CFS patients and the control group concerning CMV (*P* = .6597), EBV (*P* = .8018), and HHV‐6 (*P* = 1.00) DNA detection in PBMCs samples.

### 3.3 Prevalence of active CMV, EBV, and HHV‐6 infection

Criteria for active CMV, EBV, and HHV‐6 infection (Table 4) included presence of viral genome sequences in plasma with virus‐specific IgM class antibodies positivity and/or viral genome sequences in plasma with elevated titers of virus‐specific IgG class antibodies (R/Iv>5) without IgM antibodies.^11^ The cases positive for virus‐specific IgG class antibodies and virus DNA in PBMCs were considered as a latent viral infections.

**Table 4:** Criteria for active viral infection^a^

Viral DNA in plasma	IgM	IgG R/Iv>5
+	+	+
+	−	+
+	+	−

Abbreviations: IgG, immunoglobulin G; IgM, immunoglobulin M.

^a^ The criteria were applied for the purpose of this study.

All positive for CMV and HHV‐6 DNA plasma samples from ME/CFS cases were negative for virus‐specific IgM class antibodies, also elevated titers of virus‐specific IgG class antibodies were not found. Therefore, these cases were considered as latent viral infections (Table 5). Fourteen plasma samples from ME/CFS cases and two from the control group were positive for EBV DNA sequences. Nine of these ME/CFS cases were with elevated titers of EBV‐CA IgG class antibodies as well, two were both EBV‐CA IgM positive and with elevated titers of virus‐specific IgG class antibodies, and one was only EBV‐CA IgM positive. Considering the criteria for active infection, 12 patients with ME/CFS were estimated with active EBV infection. In the control group, one individual was regarded with active EBV infection (EBV DNA in plasma sample and elevated titers of EBV VCA IgG class antibodies). The analysis showed that the difference in the prevalence of active EBV infection between ME/CFS patients and healthy controls was statistically significant (*P* = .0027). No substantial difference in profiles of symptoms between CFS/ME patients with active and persistent EBV infection was observed.

**Table 5:** EBV, CMV, and HHV‐6 active infection estimated by nPCR and ELISA

	CFS					Controls				
	Viral DNA in plasma (n)	Viral DNA in plasma + IgM positive (n)	Viral DNA in plasma + IgG R/Iv>5 (n)	Viral DNA in plasma + IgM positive + IgG R/Iv>5 (n)	Active viral infection (n)	Viral DNA in plasma (n)	Viral DNA in plasma + IgM positive (n)	Viral DNA in plasma + IgG R/Iv>5 (n)	Viral DNA in plasma + IgM positive + IgG R/Iv>5 (n)	Active viral infection (n)
CMV	2	0	0	0	0	0	NA	NA	NA	NA
EBV	14	1	9	2	12	2	0	1	0	1
HHV‐6	1	0	0	0	0	0	NA	NA	NA	NA

Abbreviations: CFS, chronic fatigue syndrome; CMV, cytomegalovirus; EBV, Epstein‐Barr virus; ELISA, enzyme‐linked immunosorbent assay; HHV‐6, herpesvirus‐6; IgG, immunoglobulin G; IgM, immunoglobulin M; nPCR, nested polymerase chain reaction.

We assessed the number of EBV DNA copies in plasma samples of ME/CFS patients with active EBV infection by quantitative PCR. EBV DNA loads were relatively low, in the range between 790 and 1540 copies/mL of plasma samples.

## 4 DISCUSSION

Association of herpesviruses CMV, EBV, and HHV‐6 with ME/CFS has been investigated for long time, however the results are inconsistent.^9^, ^10^, ^11^, ^13^, ^15^, ^23^, ^24^, ^25^, ^26^ To contribute to the understanding of ME/CFS, in the present study, we continued these investigations and determined prevalence and type of CMV, EBV, and HHV‐6 infections among Bulgarian patients with ME/CFS. We have simultaneously tested ME/CFS patients and healthy controls for the presence of CMV, EBV, and HHV‐6 DNAs in cell‐free plasma and PBMCs samples as well for the virus‐specific antibodies.

The key finding of this study is the higher prevalence of EBV active infection (presence of viral genome sequences in plasma with EBV‐CA IgM positivity and/or viral genomic sequences in plasma with elevated titers of EBV‐CA IgG without EBV‐CA IgM) observed in patients with ME/CFS compared to the controls (*P* = .0027). EBNA‐1 IgG positivity of these cases indicated reactivation of a latent virus infection rather than primary EBV infection. At the same time, the prevalence of latent EBV infection (EBV‐CA IgG and/or PBMCs EBV DNA positivity) was high but quite similar in both ME/CFS patients and controls. Many previous studies have shown that EBV is a common trigger of ME/CFS and a possible key factors in the development of the disease. In a subset of patients with ME/CFS, the disease starts with infectious mononucleosis. In addition, altered serological profiling of the EBV immune response has been demonstrated in ME/CFS cases indicating that the immune system of some patients with ME/CFS interact with the EBV in a way different from that of healthy controls.^27^ Thus, Lerner et al^23,^, ^24^ found serum IgM class antibodies to EBV‐CA in patients with ME/CFS but not in controls and also reported elevated antibodies against EBV‐dUTPase and EBV DNA polymerase in a subset of patients with ME/CFS. Furthermore, elevated titers of early antigen (EA) IgG and antibodies to ZEBRA, a product of the immediate‐early EBV gene BZLF‐1, were detected in patients with ME/CFS.^28^, ^29^ Loebel et al^30^ in subsets of EBV‐positive ME/CFS patients have found elevated IgM response against EBV‐CA but a lack of antibodies against EBNA‐1. In a further global screening of serum antibody responses to an EBV peptide array, the same authors in serum of patients with ME/CFS revealed quite similar EBV IgG antibody response pattern as in sera of healthy controls except for the significantly enhanced IgG responses to several EBNA‐6 peptides.^25^ On the contrary, according to a recent study, no increased EBV‐CA IgG reactivity over EBNA‐1 in ME/CFS cohorts is found and EBNA‐6 peptide IgG reactivity is not significantly different between the ME/CFS and healthy control samples.^27^ In addition, some earlier studies also reported no differences in IgG titers against EBV‐CA, EBNA‐1 and EA.^10^, ^26^, ^31^ In our present study, we observed elevated EBV‐CA IgG antibodies in most of ME/CFS patients with active EBV infection. At the same time, we do not detect a significant difference in EBV‐CA IgG and IgM positivity rates between patients with ME/CFS and the control group.

There are fewеr studies on EBV DNA detection in different types of samples from patients with ME/CFS and its link to the development of ME/CFS. Thus, Loebel et al^30^ have compared EBV load in blood immune cells and found more frequently EBER DNA but not BZLF‐1 RNA in patients with ME/CFS compared to the healthy controls suggesting more frequent latent replication in ME/CFS. However, in a later study, they observed similar prevalence of EBV DNA in throat washings in patients with ME/CFS compared to healthy controls, indicating no pathogenic role of EBV reactivation in ME/CFS.^25^ At the same time, Fiore et al^32^ described a ME/CFS case with actively replicating EBV in blood.

All these conflicting results may be attributed to the methodological differences, not well‐characterized patients with ME/CFS, pathogenesis‐related EBV genetic variants, and heterogeneity of studied ME/CFS populations.^33^ Thus, heterogeneity among patients with ME/CFS is well recognized and subtypes of ME/CFS may reflect particular etiological factors.^34^ Zhang et al found evidence of subtype‐specific relationships for EBV among patients with ME/CFS analyzing EBV antibody markers in patients with ME/CFS which had been grouped into eight subtypes based on clustering of real‐time PCR expression data for 88 CFS/ME‐associated genes, 12 of them associated with EBV infection. It is assumed that heterogeneous host response to EBV reactivation could explain the heterogeneous occurrence of many of the immune and neurological abnormalities reported in patients with CFS/ME.^15^, ^35^

Our results based on serological as well as on PCR‐based techniques distinguishing between active and latent infection have also shown that there are no significant differences in the frequency of CMV and HHV‐6 active infection in patients with ME/CFS compared to the control group. We also detected high but quite similar frequency rates of CMV and HHV‐6 latent infection among both, ME/CFS and control groups. These results confirm some previous studies indicating no correlation between CMV and HHV‐6 infection and ME/CFS.^10^, ^14^, ^22^, ^26^, ^36^ At the same time, other studies indicate such a link.^37^, ^38^, ^39^ Moreover, an association between active HHV‐6 infection and ME/CFS has been demonstrated in studies distinguishing between active and latent infection using immunofluorescence assays directed against HHV‐6A antigens or early antibody assays.^40^, ^41^ Active HHV‐6 infection was detected more often in patients with ME/CFS than in controls, and this infection correlated with the occurrence of the clinical symptoms.^11^ In a recent meta‐analysis, however, we were not able to find a statistically significant difference between reported studies that have found no correlation between HHV‐6 and ME/CFS and publications that noted a correlation.^13^

The present study had some limitations including the small size of studied ME/CFS population, a potential limitation of methodology approach for estimating elevated levels of virus‐specific antibodies, the results are indicative for characterization of herpesvirus infection at a particular moment and not for the entire course of the disease.

In conclusion, this study using both serological and PCR‐based techniques for distinguishing between active and latent infection showed a high rate of active EBV infection among patients with ME/CFS indicating that at least in a subset of cases, EBV is an important factor for the development of the disease.

## CONFLICT OF INTERESTS

The authors declare that there are no conflict of interests.

## AUTHOR CONTRIBUTIONS

ES and MM conceived the idea; ES wrote the first draft of the manuscript; MM contributed towards writing and editing of the manuscript; VR collected clinical data; and AK, SR, DA, and NC carried out laboratory analyses. All authors read and approved the final manuscript.

## References

[1] Fukuda K , Straus SE , Hickie I , Sharpe MC , Dobbins JG , Komaroff A . The chronic fatigue syndrome: a comprehensive approach to its definition and study. International chronic fatigue syndrome study group. Ann Intern Med. 1994;121(12):953‐959. 7978722 10.7326/0003-4819-121-12-199412150-00009

[2] Carruthers BM . Definitions and aetiology of myalgic encephalomyelitis: how the Canadian consensus clinical definition of myalgic encephalomyelitis works. J Clin Pathol. 2007;60(2):117‐119. 16935963 10.1136/jcp.2006.042754 PMC1860613

[3] Carruthers BM , van de Sande MI , De Meirleir KL , et al. Myalgic encephalomyelitis: international consensus criteria. J Intern Med. 2011;270(4):327‐338. 21777306 10.1111/j.1365-2796.2011.02428.x PMC3427890

[4] Blomberg J , Gottfries C‐G , Elfaitouri A , Rizwan M , Rosén A . Infection elicited autoimmunity and myalgic encephalomyelitis/chronic fatigue syndrome: an explanatory model. Front Immunol. 2018;9:229 10.3389/fimmu.2018.00229 29497420 PMC5818468

[5] Glassford JAG . The neuroinflammatory etiopathology of myalgic encephalomyelitis/chronic fatigue syndrome (ME/CFS). Front Physiol. 2017;8:88 10.3389/fphys.2017.00088 28261110 PMC5314655

[6] Morris G , Maes M , Berk M , Puri BK . Myalgic encephalomyelitis or chronic fatigue syndrome: how could the illness develop? Metab Brain Dis. 2019;34:385‐415. 30758706 10.1007/s11011-019-0388-6 PMC6428797

[7] Sotzny F , Blanco J , Capelli E , et al. Myalgic encephalomyelitis/chronic fatigue syndrome—evidence for an autoimmune disease. Autoimmun Rev. 2018;17:601‐609. 10.1016/j.autrev.2018.01.009 29635081

[8] Underhill RA . Myalgic encephalomyelitis, chronic fatigue syndrome: an infectious disease. Med Hypotheses. 2015;85:765‐773. 26604026 10.1016/j.mehy.2015.10.011

[9] Ablashi DV . Viral studies of chronic fatigue syndrome. Clin Infect Dis. 1994;18(suppl 1):S130‐S133. 8148440 10.1093/clinids/18.supplement_1.s130

[10] Cameron B , Flamand L , Juwana H , et al. Serological and virological investigation of the role of the herpesviruses EBV, CMV and HHV‐6 in post‐infective fatigue syndrome. J Med Virol. 2010;82:1684‐1688. 20827765 10.1002/jmv.21873

[11] Chapenko S , Krumina A , Logina I , et al. Association of active human herpesvirus‐6,‐7 and parvovirus B19 infection with clinical outcomes in patients with myalgic encephalomyelitis/chronic fatigue syndrome. Adv Virol. 2012;2012:205085 10.1155/2012/205085 22927850 PMC3426163

[12] Komaroff AL , Cho TA . Role of infection and neurologic dysfunction in chronic fatigue syndrome. Semin Neurol. 2011;31(3):325‐337. 21964849 10.1055/s-0031-1287654

[13] Rasa S , Nora‐Krukle Z , Henning N , et al. Chronic viral infections in myalgic encephalomyelitis/chronic fatigue syndrome (ME/CFS). J Transl Med. 2018;16:268. 30285773 10.1186/s12967-018-1644-y PMC6167797

[14] Oakes B , Hoagland‐Henefield M , Komaroff AL , Erickson JL , Huber BT . Human endogenous retrovirus‐K18 superantigen expression and human herpesvirus‐6 and human herpesvirus‐7 viral loads in chronic fatigue patients. Clin Infect Dis. 2013;56:1394‐1400. 23408682 10.1093/cid/cit086

[15] Zhang L , Gough J , Christmas D , et al. Microbial infections in eight genomic subtypes of chronic fatigue syndrome/myalgic encephalomyelitis. J Clin Pathol. 2010;63:156‐164. 10.1136/jcp.2009.072561 19955554 PMC2921262

[16] Hickie I , Davenport T , Wakefield D , et al. Post‐infective and chronic fatigue syndromes precipitated by viral and non‐viral pathogens: prospective cohort study. BMJ. 2006;333:575 10.1136/bmj.38933.585764.AE 16950834 PMC1569956

[17] Cvejic E , Lemon J , Hickie IB , Lloyd AR , Vollmer‐Conna U . Neurocognitive disturbances associated with acute infectious mononucleosis, Ross River fever and Q fever: a preliminary investigation of inflammatory and genetic correlates. Brain Behav Immun. 2014;36:207‐214. 24211375 10.1016/j.bbi.2013.11.002

[18] Duvignaud A , Fianu A , Bertolotti A , et al. Rheumatism and chronic fatigue, the two facets of post‐chikungunya disease: the TELECHIK cohort study on Reunion island. Epidemiol Infect. 2018;146(5):633‐641. 29486812 10.1017/S0950268818000031 PMC5892425

[19] Saiki RK , Bugawan TL , Horn GT , Mullis KB , Erlich HA . Analysis of enzymatically amplified beta‐globin and HLA‐DQ alpha DNA with allele‐specific oligonucleotide probes. Nature. 1986;324:163‐166. 3785382 10.1038/324163a0

[20] Allen RD , Pellett PE , Stewart JA , Koopmans M . Nonradioactive PCR‐enzyme‐linked immunosorbent assay method for detection of human cytomegalovirus DNA. J Clin Microbiol. 1995;33(3):725‐728. 7751384 10.1128/jcm.33.3.725-728.1995 PMC228021

[21] Cinque P , Brytting M , Vago L , et al. Epstein‐Barr virus DNA in cerebrospinal fluid from patients with AIDS‐related primary lymphoma of the central nervous system. Lancet. 1993;342(8868):398‐401. 8101902 10.1016/0140-6736(93)92814-a

[22] Secchiero P , Carrigan DR , Asano Y , et al. Detection of human herpesvirus 6 in plasma of children with primary infection and immunosuppressed patients by polymerase chain reaction. J Infect Dis. 1995;171:273‐280. 7844362 10.1093/infdis/171.2.273

[23] Lerner AM , Beqaj SH , Deeter RG , Fitzgerald JT . IgM serum antibodies to Epstein‐Barr virus are uniquely present in a subset of patients with the chronic fatigue syndrome. In Vivo. 2004;18:101‐106. 15113035

[24] Lerner AM , Ariza ME , Williams M , et al. Antibody to Epstein‐Barr virus deoxyuridine triphosphate nucleotidohydrolase and deoxyribonucleotide polymerase in a chronic fatigue syndrome subset. PLoS One. 2012;7:e47891. 23155374 10.1371/journal.pone.0047891 PMC3498272

[25] Loebel M , Eckey M , Sotzny F , et al. Serological profiling of the EBV immune response in chronic fatigue syndrome using a peptide microarray. PLoS One. 2017;12:e0179124 10.1371/journal.pone.0179124 28604802 PMC5467847

[26] Wallace HL 2nd , Natelson B , Gause W , Hay J . Human herpesviruses in chronic fatigue syndrome. Clin Diagn Lab Immunol. 1999;6:216‐223. 10066657 10.1128/cdli.6.2.216-223.1999 PMC95690

[27] Blomberg J , Rizwan M , Böhlin‐Wiener A , et al. Antibodies to human herpesviruses in myalgic encephalomyelitis/chronic fatigue syndrome patients. Front Immunol. 2019;10:1946 10.3389/fimmu.2019.01946 31475007 PMC6702656

[28] Kawai K , Kawai A . Studies on the relationship between chronic fatigue syndrome and Epstein‐Barr virus in Japan. Intern Med. 1992;31:313‐318. 1319246 10.2169/internalmedicine.31.313

[29] Sairenji T , Yamanishi K , Tachibana Y , Bertoni G , Kurata T . Antibody responses to Epstein‐Barr virus, human herpesvirus 6 and human herpesvirus 7 in patients with chronic fatigue syndrome. Intervirology. 1995;38:269‐273. 8724857 10.1159/000150450

[30] Loebel M , Strohschein K , Giannini C , et al. Deficient EBV‐specific B‐ and T‐cell response in patients with chronic fatigue syndrome. PLoS One. 2014;9(1):e85387. 24454857 10.1371/journal.pone.0085387 PMC3893202

[31] Whelton CL , Salit I , Moldofsky H . Sleep, Epstein‐Barr virus infection, musculoskeletal pain, and depressive symptoms in chronic fatigue syndrome. J Rheumatol. 1992;19:939‐943. 1328633

[32] Fiore V , Bagella P , Peruzzu F , Caruana G , Zaru S , Mura MS . Chronic fatigue syndrome and chronic EBV infection: a long‐term challenge in a single clinical case. Infect Dis Trop Med. 2015;1(4):184.

[33] Correia S , Palser A , Elgueta Karstegl C , et al. Natural variation of Epstein‐Barr virus genes, proteins, and primary microRNA. J Virol. 2017;91(15):e00375‐17 10.1128/JVI.00375-17 28515295 PMC5512239

[34] Jason LA , Corradi K , Torres‐Harding S , Taylor RR , King C . Chronic fatigue syndrome: the need for subtypes. Neuropsychol Rev. 2005;15:29‐58. 15929497 10.1007/s11065-005-3588-2

[35] Kerr JR . Epstein‐Barr virus induced gene‐2 upregulation identifies a particular subtype of chronic fatigue syndrome/myalgic encephalomyelitis. Front Pediatr. 2019;7:59 10.3389/fped.2019.00059 30918887 PMC6424879

[36] Koelle DM , Barcy S , Huang ML , et al. Markers of viral infection in monozygotic twins discordant for chronic fatigue syndrome. Clin Infect Dis. 2002;35:518‐525. 12173124 10.1086/341774

[37] Ablashi DV , Eastman HB , Owen CB , et al. Frequent HHV‐6 reactivation in multiple sclerosis (MS) and chronic fatigue syndrome (CFS) patients. J Clin Virol. 2000;16(3):179‐191. 10738137 10.1016/s1386-6532(99)00079-7

[38] Chapenko S , Krumina A , Kozireva S , et al. Activation of human herpesviruses 6 and 7 in patients with chronic fatigue syndrome. J Clin Virol. 2006;37(suppl 1):S47‐S51. 17276369 10.1016/S1386-6532(06)70011-7

[39] Lerner AM , Beqaj SH , Deeter RG , Fitzgerald JT . IgM serum antibodies to human cytomegalovirus nonstructural gene products p52 and CM2 (UL44 and UL57) are uniquely present in a subset of patients with chronic fatigue syndrome. In Vivo. 2002;16(3):153‐159. 12182109

[40] Josephs SF , Henry B , Balachandran N , et al. HHV‐6 reactivation in chronic fatigue syndrome. Lancet. 1991;337:1346‐1347. 1674318 10.1016/0140-6736(91)93018-5

[41] Patnaik M , Komaroff AL , Conley E , Ojo‐Amaize EA , Peter JB . Prevalence of IgM antibodies to human herpesvirus 6 early antigen (p41/38) in patients with chronic fatigue syndrome. J Infect Dis. 1995;172:1364‐1367. 7594679 10.1093/infdis/172.5.1364

